# Effects of Grass-Based Crop Rotation, Nematicide, and Irrigation on the Nematode Community in Cotton

**DOI:** 10.2478/jofnem-2022-0046

**Published:** 2022-10-29

**Authors:** Lesley A. Schumacher, Zane J. Grabau, David L. Wright, Ian M. Small, Hui-Ling Liao

**Affiliations:** 1 USDA, Agricultural Research Service, Crop Genetics Research Unit, Jackson, TN 38301 Mississippi; 2 Entomology and Nematology Department, University of Florida, Gainesville, FL 32611 United States; 3 North Florida Research and Education Center, University of Florida, Quincy, FL 32351 United States

**Keywords:** bahiagrass, cotton, crop rotation, ecology, fluopyram, free-living nematodes, *Gossypium hirsutum*, irrigation, nematicide, nematode community, *Paspalum notatum*

## Abstract

Plant-parasitic and free-living nematodes – bacterivores, fungivores, omnivores, predators – comprise the nematode community. Nematicide application and crop rotation are important tools to manage plant-parasitic nematodes, but effects on free-living nematodes and nematode ecological indices need further study. The nematicide fluopyram was recently introduced in cotton (*Gossypium hirsutum*) production and its effects on the nematode community need assessment. This research was conducted in 2017 and 2018 at a long-term field site in Quincy, FL where perennial grass/sod-based (bahiagrass, *Paspalum notatum*) and conventional cotton rotations were established in 2000. The objective of this research was to evaluate the effects of fluopyram nematicide, crop rotation phase, and irrigation on free-living nematodes and nematode ecological indices based on three soil sampling dates each season. We did not observe consistent effects of crop rotation phase on free-living nematodes or nematode ecological indices. Only omnivores were consistently negatively impacted by fluopyram. Nematode ecological indices reflected this negative effect by exhibiting a degraded/ stressed environmental condition relative to untreated plots. Free-living nematodes were not negatively impacted by nematicide when sod-based rotation was used.

Nematodes are roundworms and are the most abundant group of animals on the planet, representing an estimated four out of five animals on Earth (Bongers, 1999). The nematode community contains herbivorous (plant-parasitic), mycophagous (fungivores), bacterivorous (bacterivores), omnivorous (omnivores), and predaceous (predators) nematodes. Because plant-parasitic nematodes account for $216 billion in global crop losses per year, agricultural nematology efforts focus on their management with little regard to impacts on the rest of the nematode community (Nyaku *et al*., 2017). Yet, all types of free-living nematodes (i.e., non-plant-parasitic) are found in the soil environment. The weighted proportions of these nematodes provide insight into the ecological status of an environment (Bongers and Bongers, 1998). The colonizer-persister (c-p) series is a scale ranging from cp-1 to cp-5 and assigned to nematode families based on their relative r (high fecundity, shorter life cycle) or K (low fecundity, longer life cycle) strategies (Bongers, 1990). Disturbed environments are dominated by r strategists while K strategists are often associated with more stable environments (Bongers and Bongers, 1998). Most fungivores and bacterivores have colonizer strategies and have low c-p values. Nematodes encompassing the higher c-p values include omnivorous and predaceous Dorylaimids, which have longer life cycles and lower reproduction rates than lower c-p value nematodes like bacterivorous Rhabditids (Neher, 2010).

Bongers and Ferris (1999) developed a method for evaluating ecosystem health by partitioning groups of nematodes based on their feeding strategies, which were further developed into nematode ecological indices (Ferris *et al*., 2001). Nematode ecological indices are calculated based on the weighted proportion of nematodes in the fauna that meet the index criteria (Wilson and Kakouli-Duarte, 2009). The Channel Index (CI), for instance, is a way to estimate decomposition through fungal or bacterial channels. Higher CI values indicate a tendency toward fungal decomposition channels and lower values indicate a tendency toward bacterial decomposition channels. Along with other ecological indices like the Enrichment Index (EI), Structure Index (SI), Maturity Index (MI), and Basal Index (BI), CI showcases soil health and levels of disturbance (Table 1, Bongers and Bongers, 1998; Ferris *et al*., 2001). Another important index is the MI, which measures community structure based on the abundance of colonizers and persisters (excluding plant-parasitic nematodes). It is a sensitive indicator of enrichment in agroecosystems (Bongers and Bongers, 1998). The SI measures community structure based on higher c-p nematodes in relation to number of trophic levels and ecosystem stability while the BI infers ecosystem stress (Ferris *et al*., 2001). Finally, EI measures community structure by relating enrichment nematodes against cp-1 and cp-2 bacterivores and fungivores. To assess land damage and restoration, analyzing nematode and other soil faunal communities in the context of ecosystem function has proven useful (Wilson and Kakouli-Duarte, 2009; Domene *et al*., 2011; Trap *et al*., 2016). Together with information on nematode abundances, these indices give information about the condition of the environment based on nematode community structure.

**Table 1:** Nematode ecological indices, c-p groups, and interpretation.

Index name	Abbreviation	c-p nematodes^a^	Indication (high value)^b^
Maturity	MI	1–5	Stable/enriched environment
Enrichment	EI	1–2	Enriched environment
Structure	SI	3–5	High food web complexity
Channel	CI	1–2	Fungal-dominated decomposition
Basal	BI	2	Stressed/degraded environment

a Based on weighted proportions of colonizer-persister series included in calculation of index (Bongers and Bongers, 1998). bHigh value as an indication of environmental status (Ferris *et al*., 2001). BI, Basal Index; CI, Channel Index; EI, Enrichment Index; MI, Maturity Index; SI, Structure Index.

Crop rotation is one form of cultural management and a common method used to control plant pathogens. One year of peanut (*Arachis hypogaea*) is commonly rotated with 2 yr of cotton (*Gossypium hirsutum*) in the southeastern US. A rotation using 2 yr of pasture bahiagrass (*Paspalum notatum*) rotated with 1 yr each of peanut and cotton is an alternative to the conventional rotation and known as sod-based rotation. Sod-based rotation has been shown to reduce pathogens while building organic matter, improving water infiltration, and increasing water holding capacity (Katsvairo *et al*., 2006, 2007). Crop rotation was successful in managing reniform nematode (*Rotylenchulus reniformis* Linford and Oliveira, RN), a significant pathogen of cotton (Moore and Lawrence, 2012; Khanal *et al*., 2018; Schumacher *et al*., 2020). Reducing plant-parasitic nematode population densities while maintaining population densities of free-living nematodes would be ideal so as not to disrupt important soil nutrient cycling (Griffiths and Caul, 1993; Hodson, *et al*., 2019). Free-living nematodes are considered indicators of ecosystem structure and function. Much information about soil health can be ascertained by examining these nematodes in relation to plant-parasitic nematodes and the roles they play in the soil food web. Enzyme products produced by certain fungal-feeding nematodes modify plant cell walls and alter cell metabolism (Davis *et al*., 2004). The translocation of N from the soil to the substrate via feeding activity in the form of fecal material and fungal hyphae further accelerates decomposition (Coleman and Wall, 2015). Ultimately, benefits of free-living nematodes include increasing plant-available nutrients and suppression of plant-parasitic nematodes via predatory nematodes. Assessing crop rotation impacts on the free-living nematode community is therefore of ecological importance.

Another common method of managing plant-parasitic nematodes is chemical management with the use of non-fumigant nematicides (Moore and Lawrence, 2012; Khanal *et al*., 2018; Grabau *et al*., 2021a). One such product – fluopyram – is a non-fumigant fungicide/nematicide used in crop production systems to alleviate yield losses caused by plant-parasitic nematodes (Faske and Hurd, 2015). Unfortunately, non-target impacts may be associated with the use of non-fumigant nematicides, decreasing population densities of beneficial, free-living nematodes (Yeates, 1999; Neher, 2010; Hodson *et al*., 2019; Waldo *et al*., 2019; Grabau *et al*., 2020). Fluopyram nematicide use in turfgrass resulted in negative effects on free-living nematode population densities (Waldo *et al*., 2019). Yet, in a peanut production system, fluopyram did not adversely affect free-living nematodes (Grabau *et al*., 2020). Inconsistencies in the effects of fluopyram on the nematode community showcase the importance of continued research in this area. Therefore, objectives for this study were to determine the impacts of irrigation, crop rotation phase, and fluopyram nematicide application on soil ecology based on free-living nematode population densities and ecological indices.

## Materials and Methods

### Field site and maintenance

Trials were conducted in 2017 and 2018 at a longterm research site (established in 2000) at the North Florida Research and Education Center (NFREC) in Quincy, FL (30°32.79’N, 84°35.50’W). Strip tillage was used along with an oat cover crop planted in December and terminated in March of each year. Based on prior site recommendations, a 5–15–30 (N–P–K) fertilizer was applied prior to planting each year at a rate of 280.2 kg/ha.

### Experimental design

Detailed experimental design was described by Schumacher *et al*. (2020) and is briefly summarized here. The experiment was a split–split plot arrangement with three replicates. The whole plot factor was irrigation (without or with, supplied via lateral line overhead system), subplot factor was crop rotation phase (conventional and sod-based rotation), and sub-subplot factor was without or with Velum^®^ Total nematicide (fluopyram and the insecticide imidacloprid, Bayer Crop Science, Research Triangle Park, NC), which was applied in-furrow at planting via 8002 flat fan nozzles aboard a Monosem (Monosem Co., Edwardsville, KS) two-row planter at a rate of 1.3 l/ha (0.24 kg active ingredient/ha). Sub-subplots planted to cotton (Deltapine^®^ 1646 B2XF, Bayer Crop Science, St. Louis, MO) in 2017 and 2018 were assessed, including first-year conventional cotton (C1), second-year conventional cotton (C2), and sod-based cotton (CS) crop phases. The rotations have been in place since 2000 (Zhao *et al*., 2010), and are as follows: bahiagrass–bahiagrass–peanut– cotton for the sod-based rotation; and peanut– cotton–cotton for the conventional rotation.

### Soil sampling and nematode quantification

Soil sampling was conducted in 2017 and 2018 using an Oakfield tube (Oakfield Apparatus, Oakfield, WI) sampled to a depth of approximately 30 cm in a zigzag pattern between the center two rows of each plot (Schumacher *et al*., 2020), and the nematode community was extracted from a 100-cm^3^ soil subsample using a modified sucrose-centrifugation method (Jenkins, 1964). After extraction, samples were fixed in a 2% formalin solution to preserve the nematodes and arrest nematode movement to allow for easier identification. Afterwards, the entire nematode community (plant-parasitic, fungivores, bacterivores, omnivores, and predators) was enumerated. Results from individual plant-parasitic nematode genera were reported in Schumacher *et al*. (2020). Nematodes were counted from soil samples using an inverted microscope (Carl Zeiss Inc., Thornwood, NY) and identified morphologically. Total nematode population density was recorded, the first 200 nematodes encountered were identified to genus based on keys by Bongers (1994), and Mai and Mullin (1996), and then these were adjusted to the absolute abundance per 100 cm^3^ by summing totals from each nematode genus present. Afterwards, nematode ecological indices (MI, CI, BI, SI, and EI) were calculated using the Nematode Indicator Joint Analysis (NINJA) program (Sieriebriennikov *et al*., 2014).

### Statistical analysis

Response variables were nematode population densities and ecological indices and these were analyzed separately by sampling date (preplant, midseason, and harvest) in 2017 and 2018 using a three-way split–split plot ANOVA procedure in R version 3.3.1 (The R Foundation for Statistical Computing, Vienna, Austria). Assumptions of normality were checked graphically and using Levene’s test and transformed if necessary to achieve normality (Levene, 1960; Cook and Weisburg, 1999). Significant (*P* ≤ 0.05) interactions (crop phase by nematicide) were determined from the full ANOVA model and then further analyzed by examining nematicide effects for crop phase (C1, C2, and CS). The means of variables with significant (*P* ≤ 0.05) main effects (i.e., irrigation, crop phase, or nematicide) or interactions were separated using Fisher’s least significant difference (LSD) test (*P* ≤ 0.05).

## Results

Overall, there were relatively few impacts of irrigation, crop phase, or nematicide on any of the response variables (Tables 2–7). Additionally, there were no significant main effects of irrigation, crop phase, or nematicide on predator population densities observed in any sampling date (data not shown). Significant results for individual response variables are continued below.

**Table 2:** Effects of irrigation, crop phase, and nematicide application on fungivore population density in 2017 and 2018.

	2017			2018		
	Pi^x^	Pm	Pf	Pi	Pm	Pf
Irrigation						
Irrigated	189a	150	131	236	172	256
Rainfed	103b	140	142	254	176	240
Crop phase^y^						
CS	224A	178	79B	286	153	220
C1	104B	129	81B	221	182	244
C2	110B	129	249A	228	186	281
Nematicide						
Without fluopyram	147	141	132	259	140	270
With fluopyram	144	149	141	231	208	226
ANOVA (*P*-values)						
Irrigation (I)	0.04*	0.85	0.20	0.42	0.91	0.51
Crop (C)	0.02*	0.33	<0.01**	0.50	0.75	0.84
I × C	0.48	0.26	0.45	0.32	0.63	0.96
Nematicide (N)	0.94	0.66	0.75	0.38	0.08	0.11
I × N	0.77	0.14	0.71	0.43	0.40	0.13
C × N	0.93	<0.01**	0.86	0.79	0.64	0.37
I × C × N	0.95	0.06	0.44	0.68	0.86	0.85

Different letters in columns denote means separation for significant (*P* ≤ 0.05) main effects of irrigation, crop phase, or nematicide using Fisher’s LSD test (*P* ≤ 0.05). ^x^Pi, Pm, and Pf are mean nematode population densities (per 100 cm^3^ soil) prior to planting, at midseason (52 d and 56 d after planting in 2017 and 2018), and at harvest (150 d and 151 d after planting in 2017 and 2018), respectively. ^y^C1 and C2 are first- and second-year conventional cotton, respectively, with previous 1 yr peanut; CS is sod-based cotton with previous 1 yr peanut with previous 2 yr bahiagrass. * and ** represent significant effects at *P* ≤ 0.05 and *P* ≤ 0.01, respectively. LSD, least significant difference.

**Table 3:** Effects of irrigation, crop phase, and nematicide application on bacterivore population density in 2017 and 2018.

	2017			2018		
	Pi^x^	Pm	Pf	Pi	Pm	Pf
Irrigation						
Irrigated	730	387	151	726	525	314
Rainfed	602	430	196	898	608	516
Crop phase^y^						
CS	798	431	84B	824	518	355
C1	692	333	70B	808	505	432
C2	507	461	367A	804	675	458
Nematicide						
Without fluopyram	718	433	176	828	538	414
With fluopyram	614	383	172	797	595	416
ANOVA (*P*-values)						
Irrigation (I)	0.35	0.41	0.23	0.37	0.23	0.13
Crop (C)	0.39	0.34	<0.01**	0.99	0.55	0.72
I × C	0.78	0.15	0.23	0.36	0.27	0.31
Nematicide (N)	0.14	0.27	0.93	0.80	0.55	0.98
I × N	0.21	0.67	0.79	0.29	0.86	0.13
C × N	0.31	0.02*	0.62	0.94	0.36	0.03
I × C × N	0.07	0.90	0.38	0.71	0.24	0.51

Different letters in columns denote means separation for significant (*P* ≤ 0.05) main effects of irrigation, crop phase, or nematicide using Fisher’s LSD test (*P* ≤ 0.05). ^x^Pi, Pm, and Pf are mean nematode population densities (per 100 cm^3^ soil) prior to planting, at midseason (52 d and 56 d after planting in 2017 and 2018), and at harvest (150 d and 151 d after planting in 2017 and 2018), respectively. ^y^C1 and C2 are first- and second-year conventional cotton, respectively, with previous 1 yr peanut; CS is sod-based cotton with previous 1 yr peanut with previous 2 yr bahiagrass. * and ** represent significant effects at *P* ≤ 0.05 and *P* ≤ 0.01, respectively. LSD, least significant difference.

**Table 4:** Effects of irrigation, crop phase, and nematicide application on omnivore population density in 2017 and 2018.

	2017			2018		
	Pi^x^	Pm	Pf	Pi	Pm	Pf
Irrigation						
Irrigated	19	76a	18	55	58	58
Rainfed	14	60b	12	64	50	45
Crop phase^y^						
CS	22	58	23	44	63	25
C1	22	84	9	73	38	59
C2	5	62	13	62	61	69
Nematicide						
Without fluopyram	17	87	17	80a	71a	84a
With fluopyram	15	48	13	39b	37b	19b
ANOVA (*P*-values)						
Irrigation (I)	0.59	0.04*	0.30	0.86	0.45	0.11
Crop (C)	0.18	0.67	0.17	0.63	0.59	0.53
I × C	0.40	0.97	0.63	0.35	0.73	0.91
Nematicide (N)	0.72	0.07	0.40	0.04*	<0.01**	<0.01**
I × N	0.12	0.74	0.06	0.66	0.25	0.74
C × N	0.66	0.38	0.27	0.18	0.43	<0.01**
I × C × N	0.02	0.48	0.70	0.61	0.97	0.43

Different letters in columns denote means separation for significant (*P* ≤ 0.05) main effects of irrigation, crop phase, or nematicide using Fisher’s LSD test (*P* ≤ 0.05). ^x^Pi, Pm, and Pf are mean values of nematode ecological indices prior to planting, at midseason (52 d and 56 d after planting in 2017 and 2018), and at harvest (150 d and 151 d after planting in 2017 and 2018), respectively. ^y^C1 and C2 are first- and second-year conventional cotton, respectively, with previous 1 yr peanut; CS is sod-based cotton with previous 1 yr peanut with previous 2 yr bahiagrass. * and ** represent significant effects at *P* ≤ 0.05 and *P* ≤ 0.01, respectively. MI, Maturity Index; LSD, least significant difference.

**Table 5:** Effects of irrigation, crop phase, and nematicide application on BI in 2017 and 2018.

	2017			2018		
	Pi^x^	Pm	Pf	Pi	Pm	Pf
Irrigation						
Irrigated	30	28b	38	35	37	36
Rainfed	26	33a	43	42	42	43
Crop phase^y^						
CS	22B	25	32	37	35	41
C1	21B	28	40	35	38	40
C2	41A	38	50	44	46	37
Nematicide						
Without fluopyram	25	30	38	36	39	37
With fluopyram	31	30	44	41	40	42
ANOVA (*P*-values)						
Irrigation (I)	0.32	0.05*	0.62	0.28	0.31	0.44
Crop (C)	0.03*	0.11	0.16	0.40	0.30	0.85
I × C	0.66	0.85	0.66	0.22	0.90	0.98
Nematicide (N)	0.15	0.98	0.29	0.25	0.84	0.34
I × N	0.69	0.87	0.15	0.66	0.70	0.30
C × N	0.24	0.29	0.06	0.92	0.01**	0.24
I × C × N	0.35	0.55	0.46	0.37	0.65	0.84

Different letters in columns denote means separation for significant (*P* ≤ 0.05) main effects of irrigation, crop phase, or nematicide using Fisher’s LSD test (*P* ≤ 0.05). ^x^Pi, Pm, and Pf are mean values of nematode ecological indices prior to planting, at midseason (52 d and 56 d after planting in 2017 and 2018), and at harvest (150 d and 151 d after planting in 2017 and 2018), respectively. ^y^C1 and C2 are first- and second-year conventional cotton, respectively, with previous 1 yr peanut; CS is sod-based cotton with previous 1 yr peanut with previous 2 yr bahiagrass. * and ** represent significant effects at *P* ≤ 0.05 and *P* ≤ 0.01, respectively. LSD, least significant difference; BI, Basal Index.

**Table 6:** Effects of irrigation, crop phase, and nematicide application on EI in 2017 and 2018.

	2017			2018		
	Pi^x^	Pm	Pf	Pi	Pm	Pf
Irrigation						
Irrigated	66	56	46	53	46	48
Rainfed	71	53	43	40	38	47
Crop phase^y^						
CS	76A	65A	45	52	47	48
C1	75A	58AB	43	47	44	44
C2	54B	40B	45	40	34	51
Nematicide						
Without fluopyram	70	54	46	49	38	47
With fluopyram	67	54	43	44	45	48
ANOVA (*P*-values)						
Irrigation (I)	0.30	0.71	0.75	0.16	0.25	0.98
Crop (C)	0.02*	0.01**	0.99	0.34	0.13	0.55
I × C	0.74	0.99	0.64	0.12	0.24	0.72
Nematicide (N)	0.40	0.98	0.61	0.32	0.07	0.72
I × N	0.77	0.88	0.88	0.51	0.92	0.51
C × N	0.43	0.91	0.38	0.63	0.02*	0.58
I × C × N	0.23	0.91	0.68	0.17	0.92	0.19

Different letters in columns denote means separation for significant (*P* ≤ 0.05) main effects of irrigation, crop phase, or nematicide using Fisher’s LSD test (*P* ≤ 0.05). ^x^Pi, Pm, and Pf are mean values of nematode ecological indices prior to planting, at midseason (52 d and 56 d after planting in 2017 and 2018), and at harvest (150 d and 151 d after planting in 2017 and 2018), respectively. ^y^C1 and C2 are first- and second-year conventional cotton, respectively, with previous 1 yr peanut; CS is sod-based cotton with previous 1 yr peanut with previous 2 yr bahiagrass. * and ** represent significant effects at *P* ≤ 0.05 and *P* ≤ 0.01, respectively. EI, Enrichment Index; LSD, least significant difference.

**Table 7:** Effects of irrigation, crop phase, and nematicide application on MI in 2017 and 2018.

	2017			2018		
	Pi^x^	Pm	Pf	Pi	Pm	Pf
Irrigation						
Irrigated	1.84	2.23	2.33	2.09	2.19	2.25
Rainfed	1.71	2.17	2.24	2.13	2.21	2.08
Crop phase^y^						
CS	1.71	2.09	2.46	2.02	2.21	2.09
C1	1.73	2.27	2.45	2.17	2.19	2.24
C2	1.88	2.23	1.97	2.13	2.20	2.16
Nematicide						
Without fluopyram	1.77	2.25	2.31	2.12	2.26a	2.27a
With fluopyram	1.78	2.15	2.25	2.09	2.14b	2.06b
ANOVA (*P*-values)						
Irrigation (I)	0.17	0.07	0.06	0.75	0.44	0.16
Crop (C) I × C	0.23 0.84	0.07 0.91	0.16 0.90	0.25 0.73	0.95 0.59	0.58 0.81
Nematicide (N)	0.84	0.31	0.93	0.41	0.02*	<0.01**
I × N	0.67	0.83	0.08	0.27	0.77	0.35
C × N	0.45	0.28	0.61	0.08	0.30	0.01**
I × C × N	0.14	0.80	0.64	0.17	0.61	0.22

Different letters in columns denote means separation for significant (*P* ≤ 0.05) main effects of irrigation, crop phase, or nematicide using Fisher’s LSD test (*P* ≤ 0.05). ^x^Pi, Pm, and Pf are mean nematode population densities (per 100 cm^3^ soil) prior to planting, at midseason (52 d and 56 d after planting in 2017 and 2018), and at harvest (150 d and 151 d after planting in 2017 and 2018), respectively. ^y^C1 and C2 are first- and second-year conventional cotton, respectively, with previous 1 yr peanut; CS is sod-based cotton with previous 1 yr peanut with previous 2 yr bahiagrass. * and ** represent significant effects at *P* ≤ 0.05 and *P* ≤ 0.01, respectively. LSD, least significant difference.

## Fungivores and bacterivores

**Figure 1:**
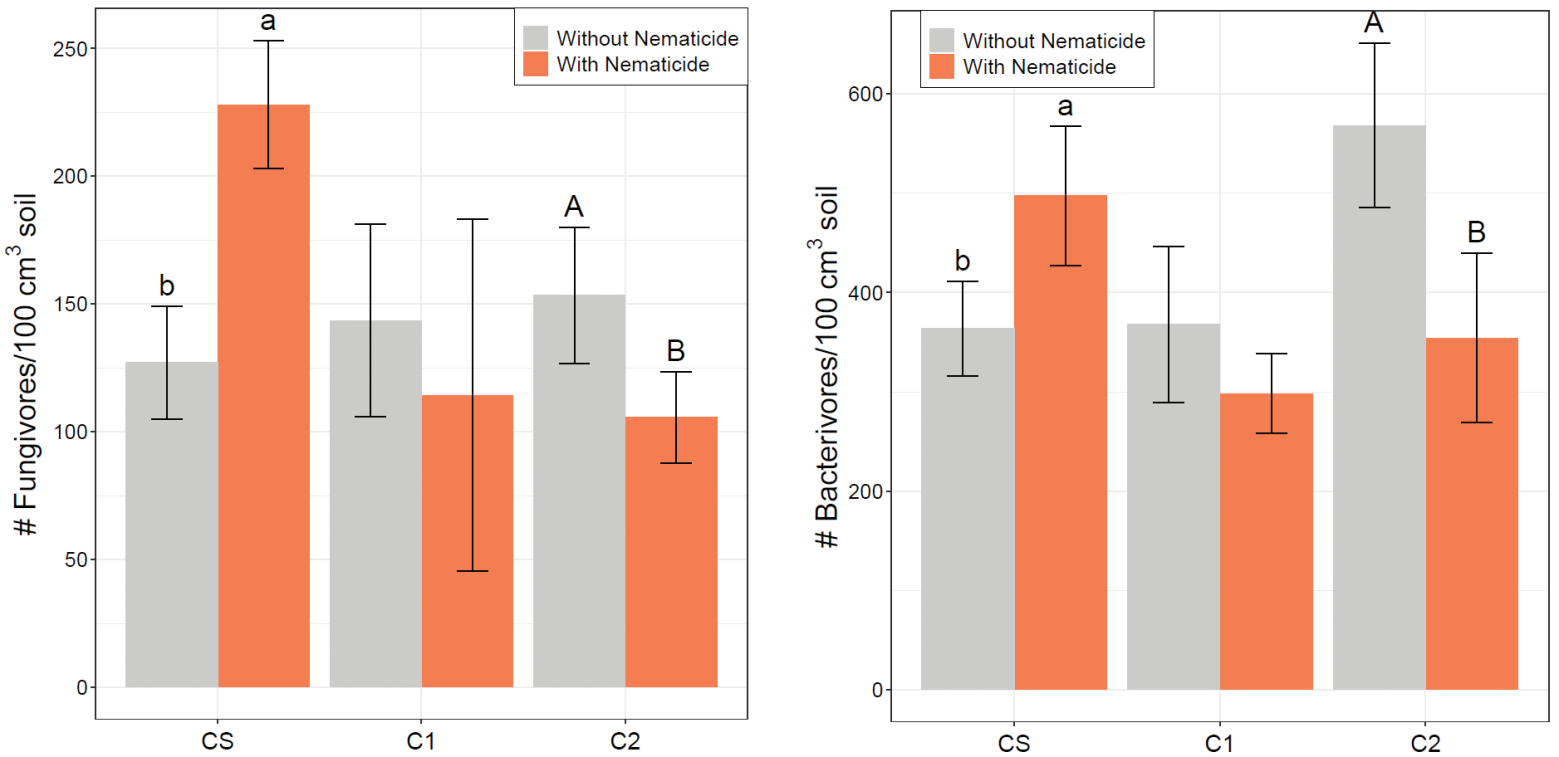
Fungivore (left) and bacterivore (right) population density (nematodes/100 cm^3^ soil) in midseason 2017 soil samples. CS is the cotton phase of the sod-based rotation, C1 is first-year conventional cotton, and C2 is second-year conventional cotton. Without Nematicide and With Nematicide refer to absence or presence of fluopyram, respectively. Different letters denote significant differences between nematicide treatments within crop phase (Fisher’s LSD, *P* ≤ 0.05). LSD, least significant difference.

**Figure 2:**
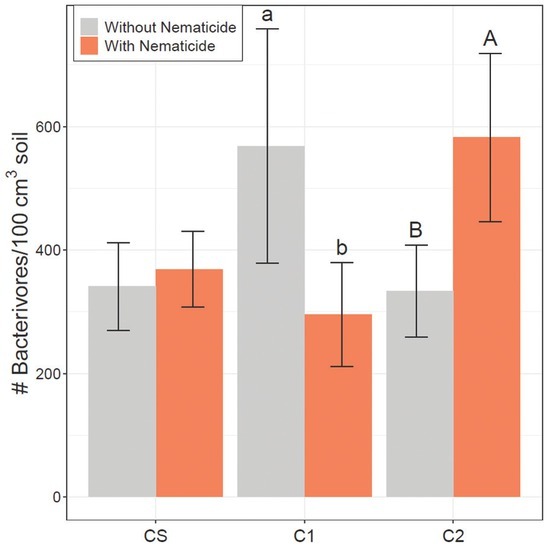
Bacterivore population density (nematodes/100 cm^3^ soil) in harvest 2018 (right) soil samples. CS is the cotton phase of the sod-based rotation, C1 is first-year conventional cotton, and C2 is second-year conventional cotton. Without Nematicide and With Nematicide refer to absence or presence of fluopyram, respectively. Different letters denote significant differences between nematicide treatments within crop phase (Fisher’s LSD, *P* ≤ 0.05). LSD, least significant difference.

In preplant 2017 soil samples, fungivore population density was significantly greater in irrigated plots than rainfed plots, but irrigation did not significantly affect fungivores in any other season (Table 2). Fungivore population density was greater in preplant CS plots than conventional cotton plots in preplant 2017 soil samples (Table 2). Both fungivore and bacterivore populations were greater in C2 in harvest 2017 soil samples (Tables 2 and 3, respectively). There were significant rotations by nematicide interactions for both fungivores and bacterivores in midseason 2017 (Tables 2 and 3) as fungivores and bacterivores were both greater in nematicide-treated plots of CS in midseason 2017 soil samples (Fig. 1). However, bacterivores and fungivores were both greater in untreated plots of C2 than plots with nematicide in midseason 2017. There was also a significant interaction in harvest 2018 for bacterivores (Table 3), and bacterivore abundances were greater in untreated plots of C1 and greater in nematicide-treated plots of C2 (Fig. 2).

### Omnivores

**Figure 3:**
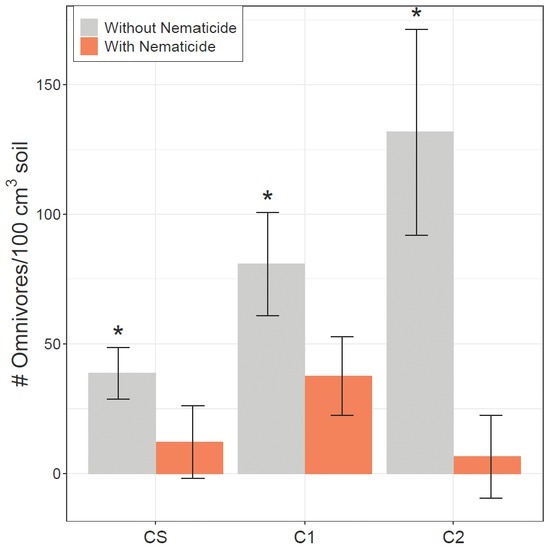
Omnivore population density (nematodes/100 cm^3^ soil) in harvest 2018 soil samples. CS is the cotton phase of the sod-based rotation, C1 is first-year conventional cotton, and C2 is second-year conventional cotton. Without Nematicide and With Nematicide refer to absence or presence of fluopyram, respectively. * indicates significant nematicide effect within the given crop (Fisher’s LSD, *P* ≤ 0.05). LSD, least significant difference.

Omnivore population density was greatest in irrigated plots and significantly lower in rainfed plots in midseason 2017 soil samples (Table 4). Untreated plots had significantly more omnivores than nematicide-treated plots in preplant 2018, midseason 2018, and harvest 2018 soil samples (Table 4). There was a nematicide by crop rotation interaction in harvest 2018, but nematicide significantly decreased omnivore population density in each crop phase in harvest 2018 soil samples (Fig. 3).

### Basal Index

**Figure 4:**
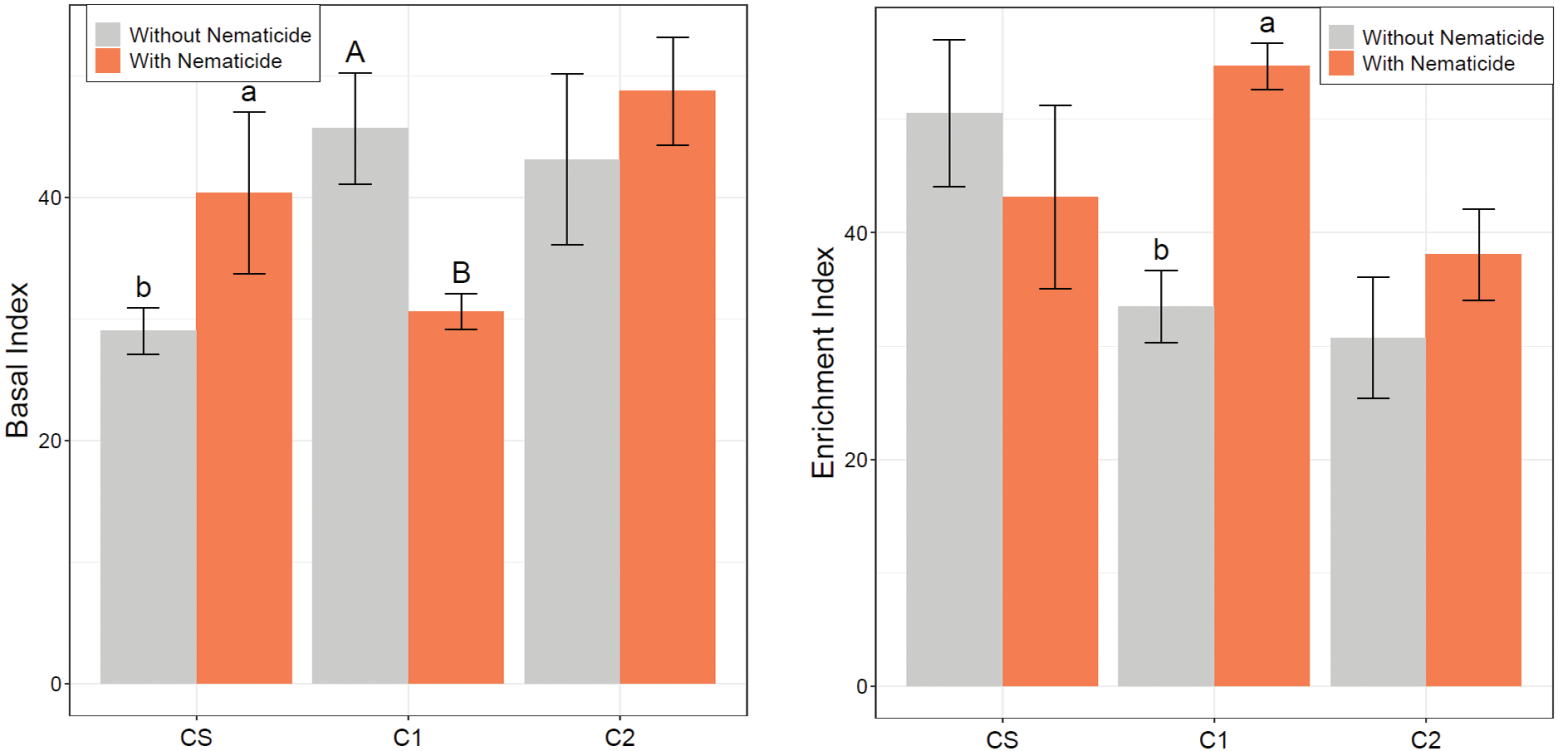
Basal Index (left) and EI (right) based on midseason 2018 soil samples. CS is the cotton phase of the sod-based rotation, C1 is first-year conventional cotton, and C2 is second-year conventional cotton. Without Nematicide and With Nematicide refer to absence or presence of fluopyram, respectively. Different letters denote significant differences between nematicide treatments within crop phase (Fisher’s LSD, *P* ≤ 0.05). EI, Enrichment Index; LSD, least significant difference.

In 2017 preplant soil samples, C2 plots had a significantly greater BI value than CS or C1 plots (Table 5). In midseason 2017 soil samples, rainfed plots had a significantly greater BI value than irrigated plots (Table 5). In midseason 2018 soil samples, nematicide effects on BI were significant in CS and C1, but not C2 (Table 5 and Fig. 4). In CS, BI was greatest in nematicide-treated plots and significantly lower in untreated plots. In C1, however, the opposite trend was observed where BI was greatest in untreated plots and significantly lower in nematicide-treated plots.

### Enrichment Index

Preplant CS and C1 plots had significantly greater EI values than preplant C2 plots in preplant 2017 soil samples (Table 6). EI was significantly affected by crop phase in midseason 2017 soil samples, where EI was greatest in CS and least in C2 (Table 6). There was a significant crop phase by nematicide interaction for EI in midseason 2018 soil samples (Fig. 4). Nematicide effects were significant in C1, but not in CS or C2. In C1, EI was greatest in nematicide-treated plots and significantly lower in untreated plots.

### Maturity, channel, and structure indices

**Figure 5:**
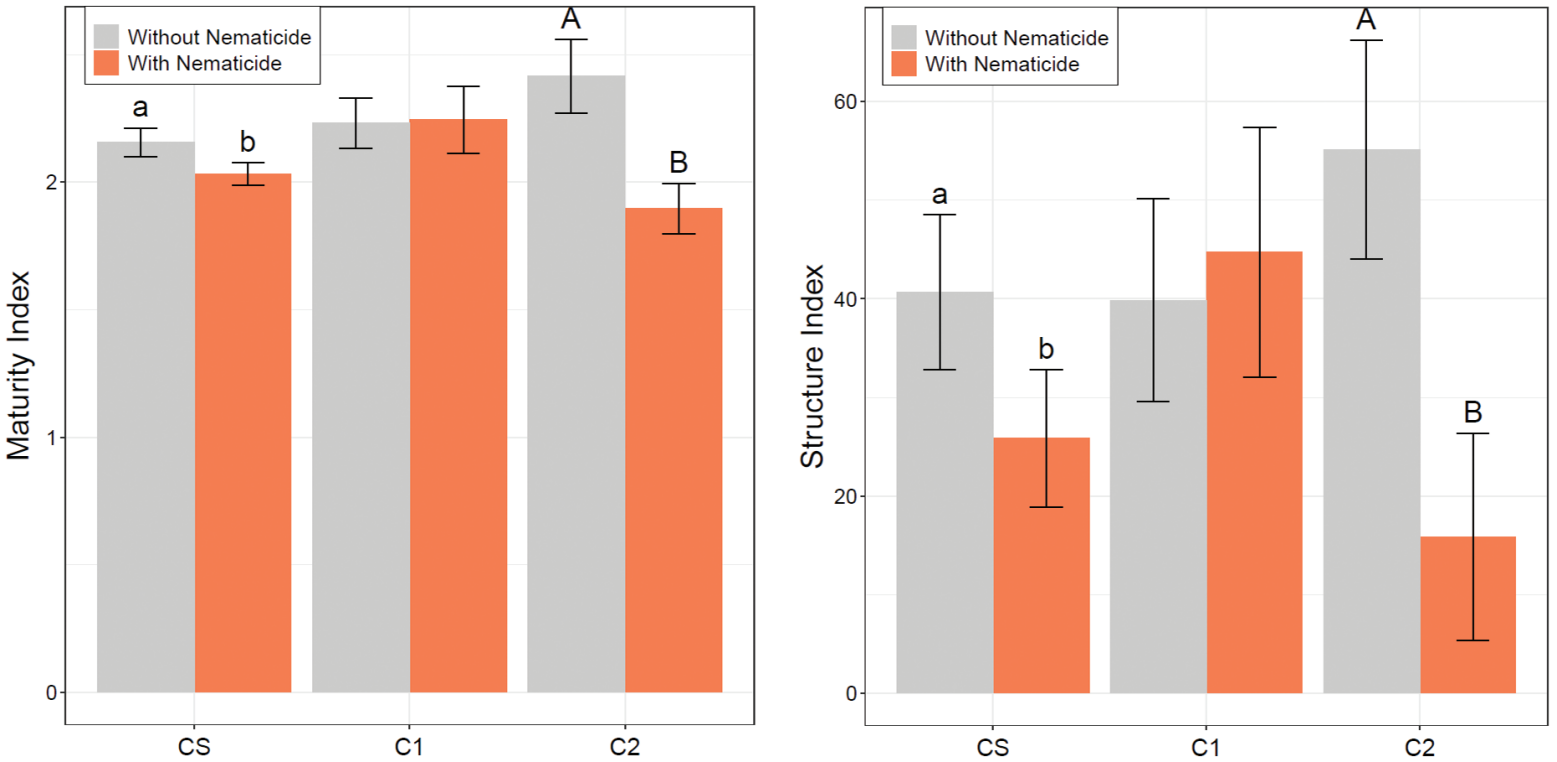
Maturity Index (left) and SI (right) based on harvest 2018 soil samples. CS is the cotton phase of the sod-based rotation, C1 is first-year conventional cotton, and C2 is second-year conventional cotton. Without Nematicide and With Nematicide refer to absence or presence of fluopyram, respectively. Different letters denote significant differences between nematicide treatments within crop phase (Fisher’s LSD, *P* ≤ 0.05). LSD, least significant difference; SI, Structure Index.

Nematicide-treated plots had a lower MI value than untreated plots in both midseason 2018 and harvest 2018 soil samples (Table 7). Nematicide effects on MI were significant in CS and C2, but not C1 in harvest 2018 soil samples. In CS and C2, MI was greatest in untreated plots and significantly lower in nematicide-treated plots. Nematicide-treated plots had a significantly lower CI value than untreated plots (39 and 57, respectively) in harvest 2018 soil samples (*P* = 0.03). In harvest 2018 soil samples, nematicide effects on SI were significant in CS and C2, but not C1 (Fig. 5, *P* < 0.01). In CS and C2, SI was greatest in untreated plots and significantly lower in nematicide-treated plots.

## Discussion

Nematodes are important indicator taxa and assessment of nematode communities may enhance understanding of global distribution patterns and help in predicting impacts of various agricultural management practices and ecosystem health (Bongers and Ferris, 1999; Cesarz *et al*., 2019; Van den Hoogen *et al*., 2019). Maintaining populations of free-living nematodes while reducing populations of plant-parasitic nematodes is a long-term goal of nematode management (Neher *et al*., 2019). Free-living nematodes are important in soil nutrient cycling and beneficial to crop production (Griffiths and Caul, 1993; Davis *et al*., 2004; Neher, 2010; Coleman and Wall, 2015). Goals for this research were to assess effects of irrigation, crop rotation phase, and non-fumigant nematicide application on the nematode community in cotton.

Previous research on irrigation showed that plant-parasitic nematode population densities were greater during periods of reduced precipitation/irrigation (Bird *et al*., 2009). We observed the opposite trend in a few instances in our study, yet overall irrigation did not have a consistent impact on free-living nematodes. Additionally, nematode ecological indices were rarely influenced by irrigation.

Tracking differences in nematode community structure in sod-based and conventional cotton provided insight on how free-living nematodes responded to different crop rotation phases. In our study, we did not observe consistent effects on any response variable as none were affected in both years of our study and only a few had effects in multiple seasons within a single year. Although fungivores were affected by crop phase in more sampling dates than bacterivores, neither group was consistently affected by crop phase. Similarly, neither omnivores nor predators were affected by crop phase. Rotation was expected to affect trophic groups, particularly microbe feeders like bacterivores as they thrive on simple resources with low C:N ratios whereas fungivores thrive on recalcitrant resources that are more difficult to break down (Neher, 2010). Hou *et al*. (2010) showed that fallow fields have high numbers of fungivores. To this end, after 2 yr in bahiagrass in the sod-based rotation, more recalcitrant resources were likely present in the sod-based cotton phase. Since bahiagrass is associated with large amounts of mycorrhizae (Sylvia *et al*., 2001) and increased complexity in soil trophic interactions (Zhang *et al*., 2022), it is surprising that crop phase had inconsistent effects on fungivores and bacterivores.

It is evident that the type of crop is a driver for the associated free-living nematode community. For instance, long-term corn promoted a mature, fungal-based ecosystem while long-term soybean promoted a more disturbed, enriched, bacterial-based system (Grabau and Chen, 2016). Grabau *et al*. (2021b) found that in a conventional horticulture production system, nematode trophic groups were less abundant than in an organic system. This may explain why the results from our study yielded fewer differences between conventional and sod-based systems, since both used the same levels of site management regarding inputs. Additionally, Matute and Anders (2012) found that in a rice–soybean–corn rotation, rotations including soybean significantly increased all trophic groups of soil nematode populations while rice and corn had reductive effects. Unlike sod-based rotations, which were characterized by decreasing reniform and increasing spiral and ring nematode population densities, free-living nematodes were less impacted by crop phase in the same rotations (Schumacher *et al*., 2020).

High MI or EI values indicate a stable and/ or enriched environment, high SI values indicate high food web complexity, high CI values indicate fungal-dominated decomposition pathways, and high BI values indicate stressed and/or degraded environments (Ferris *et al*., 2001). Crop rotation had very little impact on nematode community indices. In 1 yr, EI values were often lowest in second-year conventional cotton, but this was not consistent across seasons. A lower EI value indicates a less enriched environment based on lower c-p value nematode taxa (i.e., bacterivores and fungivores), meaning there were less resources available in the second-year conventional cotton phase (Bongers and Bongers, 1998). Our results differ from other studies, where crop rotation did affect the nematode community. For instance, Neher *et al*. (2019) showed that crop rotation had a more pronounced effect on nematode community indices than biocides, increasing CI and EI values. MI and SI experienced the same trend where nematicide significantly reduced their values compared to untreated plots in both CS and C2. The recent crops being similar may have limited differences in this study as both CS and C1 follow peanut and only cotton phases were assessed. More diverse cropping systems may have warranted greater or more consistent differences in free-living nematode population densities.

Fluopyram nematicide had minimal impacts on free-living nematode population densities, aside from omnivores. None of the nematicide effects were consistent across seasons, especially regarding nematicide effects under individual crop phases. Hodson *et al*. (2019) showed that population densities of fungivores were more adversely affected by nematicide than population densities of bacterivores, whereas in our study, effects of nematicide application were inconsistent, where in some sampling dates fungivores and bacterivores were negatively impacted by its application in the conventional rotation. This is indicative that nematicide application in C2 did negatively impact free-living nematodes while in CS there were no negative impacts on these free-living nematode populations. The observed significant crop by nematicide interaction in harvest 2018 may indicate that omnivores were preferentially feeding on cotton, as their population densities were greatest in second-year conventional cotton plots. Interestingly, there was no consistent trend in crop phase on omnivore population densities.

If environmental disturbance can be defined in the context of applying agricultural chemicals, such as nematicides, then nematodes that serve as environmental indicators are useful to study ecological effects. In general, nematode populations decrease after agricultural chemical management (Desaeger *et al*., 2020). Perhaps due to increased microbial activity in soil following nematicide application, populations of lower c-p nematodes may rebound quickly (Coleman and Wall, 2015). Carrascosa *et al*. (2014) found that nematode community structure was significantly affected in fumigated soil where free-living populations ultimately increased and shifted to a more disturbed environment. Waldo *et al*. (2019) found that nematicides significantly decreased free-living nematode population densities. Fluopyram was shown to impact plant-parasitic nematode genera differently in a sod-based rotation, increasing spiral and reniform nematodes while having no effect on ring nematodes (Schumacher *et al*., 2020), and so it is not surprising that it impacted free-living nematodes in different ways in the present study. Omnivores, which are higher c-p nematodes, appeared to be sensitive to fluopyram nematicide application. In 2018, we observed very consistent trends regarding fluopyram nematicide application where omnivore population densities were always lower in nematicide-treated plots. Another study showed that nematicides were highly toxic to omnivores in a corn production system (Smolik, 1983). Dissimilarly, omnivores were not significantly affected by fluopyram application in a peanut production system (Grabau *et al*., 2020). Our results show that omnivore abundances and several nematode ecological indices were negatively affected by nematicide application in cotton. It is possible that after 2 yr with nematicide application we observed a negative residual ecological effect in the soil (Hodson *et al*., 2019). Predator population densities were very low. This made it difficult to assess treatment effects on their populations.

Generally, nematode communities with greater population densities of omnivores and predators (i.e., higher c-p nematodes) have higher MI values (Hodson *et al*., 2019). Food web complexity is reflected by the SI, and so higher values will also indicate a more complex food web (Ferris *et al*., 2001). Nematode communities that are dominated by lower c-p nematodes will have a higher EI value, indicative of resource availability and detrital consumer activity (Ferris *et al*., 2001; Hodson *et al*., 2019). Lastly, the lowest c-p nematode groups (i.e., 1 and 2) are represented by the CI, which indicates whether decomposition is occurring via bacterial or fungal channels (Ferris *et al*., 2001; Hodson *et al*., 2019). In this study, we observed trends similar to those reported by Hodson *et al*. (2019) in that nematode community structure under non-fumigant nematicide application was affected similarly to fumigant nematicide application.

Nematicide application negatively impacted certain free-living nematodes in the conventional rotation. The underlying reason for sensitivity to nematicides is due to the permeable nematode cuticle, which can come into direct contact with pollutants (Yeates, 1999; Neher, 2010; Hodson *et al*., 2019). Prior research found that following fumigation, omnivore and predator populations decreased, which led to a lower SI value (Hodson *et al*., 2019). Except for one sampling date, our results did not support this, perhaps because we used a non-fumigant nematicide. Furthermore, MI was significantly affected by nematicide application in each crop phase in two sampling dates during 2018, indicating differing degrees of ecological succession based on a rotation system. These results differed from those in other studies, where fumigant nematicide application did not affect MI in an almond orchard system (Hodson *et al*., 2019). Due to its longer half-life, fluopyram remains active in the soil for months whereas soil fumigants only remain active for days (Wei *et al*., 2016). Yet, fluopyram has shown inconsistent results in managing plant-parasitic nematodes in cotton (Schumacher *et al*., 2020; Grabau *et al*., 2021a).

Other integrated management systems have seen varied effects on the nematode community. The link between nematode community diversity and ecological processes is still unclear, with several attempts to elucidate these concepts made in past research (Schafer, 1973; Rosenberg, 1976; Ettema, 1998; Neher and Darby, 2006). Our results were inconsistent with other research assessing management effects on the nematode community based on MI values. Porazinska *et al*. (1999) noted that lower MI values in a citrus soil ecosystem resulted from suppression of omnivores due to agroecosystem inputs (i.e., mulch) over time. In a study assessing fumigant effects on free-living nematodes in an almond orchard, no significant differences in MI were observed between treatments within individual years (Hodson *et al*., 2019). However, because crop phase was a factor in our study, this may explain why we observed significant MI values. The higher MI values in untreated plots encountered in the second year of our study indicate a more stable environment, in comparison to nematicide-treated plots that may have been less stable. Some of the significant nematicide effects on nematode ecological indices may be explained by the prolonged exposure to fluopyram application in the plots for three consecutive cropping seasons. Grabau and Chen (2016) reported that granular nematicide treatments in a long-term corn–soybean rotation disturbed the soil food web. Similarly, our results suggest a long-term effect of fluopyram on nematode ecology in sod-based and conventional crop rotation systems, reflected by significant results observed on nematode ecological indices in harvest 2018 soil samples.

## Conclusion

This study highlighted the importance of understanding how plant-parasitic nematode management practices (i.e., irrigation, crop rotation phase, and nematicide application) affect non-target, free-living nematodes. Assessing the effects of sustainable farming practices, like sod-based rotation, can therefore be accomplished by examining these nematode communities. Overall, none of the factors consistently affected the nematode community. Because our rotations utilized the same crop (i.e., cotton), perhaps less overall differences in nematode community structure were observed than in other crop rotation studies. In our study, omnivores were more sensitive to environmental disturbance in terms of nematicide application. However, nematode ecology was not consistently influenced by nematicide application in sod-based and conventional crop rotation systems. Fluopyram nematicide had a negative impact on omnivores, but minimal impact on the rest of the nematode community, regardless of crop rotation phase. Free-living nematodes were not negatively impacted by nematicide when sod-based rotation was used. This supports the idea that nematicide application can be reduced in sod-based rotation while not adversely affecting lower c-p nematodes.

## References

[j_jofnem-2022-0046_ref_001] Bird G. W., Schumacher L. A, Davenport, J., Kendle T. (2009). Influence of precipitation on populations of Heterodera glycines in the presence and absence of resistant cultivars. Journal of Nematology.

[j_jofnem-2022-0046_ref_002] Bongers T. (1990). The maturity index: An ecological measure of environmental disturbance based on nematode species composition. Oecologia.

[j_jofnem-2022-0046_ref_003] Bongers T. (1994). De nematoden van Nederland. Pirola, Schoorl.

[j_jofnem-2022-0046_ref_004] Bongers T. (1999). The maturity index, the evolution of nematode life history traits, adaptive radiation and cp-scaling. Plant and Soil.

[j_jofnem-2022-0046_ref_005] Bongers T., Bongers M. (1998). Functional diversity of nematodes. Applied Soil Ecology.

[j_jofnem-2022-0046_ref_006] Bongers T., Ferris H. (1999). Nematode community structure as a bioindicator in environmental monitoring. Trends Ecology Evolution.

[j_jofnem-2022-0046_ref_007] Carrascosa M., Sánchez-Moreno S., Alonso-Prados J. L. (2014). Relationships between nematode diversity, plant biomass, nutrient cycling and soil suppressiveness in fumigated soils. European Journal of Soil Biology.

[j_jofnem-2022-0046_ref_008] Cesarz S., Schulz A. E., Beugnon R., Eisenhauer N. (2019). Testing soil nematode extraction efficiency using different variations of the Baermann-funnel method. Soil Organisms.

[j_jofnem-2022-0046_ref_009] Coleman D. C., Wall D. H., Paul E. A. (2015). Soil microbiology, ecology, and biochemistry.

[j_jofnem-2022-0046_ref_010] Cook R. D., Weisburg S. (1999). Response transformations. Applied regression including computing and graphics.

[j_jofnem-2022-0046_ref_011] Davis E. L., Hussey R. S., Baum T. J. (2004). Getting to the roots of parasitism. Trends in Parasitology.

[j_jofnem-2022-0046_ref_012] Desaeger J., Wram C., Zasada I. (2020). New reduced-risk agricultural nematicides – Rationale and review. Journal of Nematology.

[j_jofnem-2022-0046_ref_013] Domene X., Chelinho S., Campana P., Natal-da-Luz T., Alcañiz J. M., Andrés P., Römbke J., Sousa J. P. (2011). Influence of soil properties on the performance of Folsomia candida: Implications for its use in soil ecotoxicology testing. Environmental Toxicology and Chemistry.

[j_jofnem-2022-0046_ref_014] Ettema C. H. (1998). Soil nematode diversity: Species coexistence and ecosystem function. Journal of Nematology.

[j_jofnem-2022-0046_ref_015] Faske T. R., Hurd K. (2015). Sensitivity of Meloidogyne incognita and Rotylenchulus reniformis to fluopyram. Journal of Nematology.

[j_jofnem-2022-0046_ref_016] Ferris H., Bongers T., de Goede R. G. M. (2001). A framework for soil food web diagnostics: Extension of the nematode faunal analysis concept. Applied Soil Ecology.

[j_jofnem-2022-0046_ref_017] Grabau Z. J., Chen S. (2016). Influence of longterm corn-soybean crop sequences on soil ecology as indicated by the nematode community. Applied Soil Ecology.

[j_jofnem-2022-0046_ref_018] Grabau Z. J., Liu C., Schumacher L. A., Small I. M., Wright D. L. (2021a). In-furrow fluopyram nematicide efficacy for Rotylenchulus reniformis management in cotton production. Crop Protection.

[j_jofnem-2022-0046_ref_019] Grabau Z. J., Treadwell D. D., Perez Orozco J. J., Campbell D. N., Hochmuth R. C. (2021b). Organic or conventional production system and nutrient rate affect the nematode community in carrot production. Journal of Nematology.

[j_jofnem-2022-0046_ref_020] Grabau Z. J., Mauldin M. D., Habteweld A., Carter E. T. (2020). Nematicide efficacy at managing Meloidogyne arenaria and non-target effects on free-living nematodes in peanut production. Journal of Nematology.

[j_jofnem-2022-0046_ref_021] Griffiths B. S., Caul S. (1993). Migration of bacterial-feeding nematodes, but not protozoa, to decomposing grass residues. Biology and Fertility of Soils.

[j_jofnem-2022-0046_ref_022] Hodson A. K., Milkereit J., John G. C., Doll D. A., Duncan R. A. (2019). The effect of fumigation on nematode communities in California almond orchards. Nematology.

[j_jofnem-2022-0046_ref_023] Hou X., Hu N., Zhang X., Liang L., Zhai R. (2010). Vertical distribution of soil nematode communities under different tillage systems in lower reaches of Liaohe river. Chinese Geographical Science.

[j_jofnem-2022-0046_ref_024] Jenkins W. R. (1964). A rapid centrifugal-flotation technique for separating nematodes from soil. Plant Disease Reporter.

[j_jofnem-2022-0046_ref_025] Katsvairo T.W., Wright D. L., Marois J. J., Hartzog D. L., Rich J. R. (2007). Performance of peanut and cotton in a bahiagrass cropping system. Agronomy Journal.

[j_jofnem-2022-0046_ref_026] Katsvairo T. W., Wright D. L., Marois J. J., Hartzog D. L., Rich J. R., Wiatrak P. J. (2006). Sod-livestock integration into the peanut-cotton rotation: A systems farming approach. Agronomy Journal.

[j_jofnem-2022-0046_ref_027] Khanal C., McGawley E. C., Overstreet C., Stetina S. R. (2018). The elusive search for reniform nematode resistance in cotton. Phytopathology.

[j_jofnem-2022-0046_ref_028] Levene H., Olkin I. (1960). Contributions to probability and statistics.

[j_jofnem-2022-0046_ref_029] Linford M. B., Oliveira J. M. (1940). Rotylenchulus reniformis, Nov.gen. n. sp., a nematode parasite of roots. Proceedings of Helminthological Society Washington.

[j_jofnem-2022-0046_ref_030] Mai W. F., Mullin P. G. (1996). Plant-parasitic nematodes: A pictorial key to genera.

[j_jofnem-2022-0046_ref_031] Matute M. M., Anders M. (2012). Influence of rice rotation systems on soil nematode trophic groups in Arkansas. Journal of Agricultural Science.

[j_jofnem-2022-0046_ref_032] Moore S. R., Lawrence K. S. (2012). Rotylenchulus reniformis in cotton: Current methods of management and the future of site-specific management. Nematropica.

[j_jofnem-2022-0046_ref_033] Neher D. A. (2010). Ecology of plant and free-living nematodes in natural and agricultural soil. Annual Review of Phytopathology.

[j_jofnem-2022-0046_ref_034] Neher D. A., Darby B. J., Abebe E., Andrassy I., Transpurger W. (2006). Freshwater nematodes: Ecology and taxonomy.

[j_jofnem-2022-0046_ref_035] Neher D. A., Nishanthan T., Grabau Z. J., Chen S. Y. (2019). Crop rotation and tillage affect nematode communities more than biocides in monoculture soybean. Applied Soil Ecology.

[j_jofnem-2022-0046_ref_036] Nyaku S. T., Affokpon A., Danquah A., Brentu F. C., Shah M. M., Mahamood M. (2017). Nematology – Concepts, diagnosis, and control.

[j_jofnem-2022-0046_ref_037] Porazinska D. L., Duncan L. W., McSorley R., Graham J. H. (1999). Nematode communities as indicators of status and processes of a soil ecosystem influenced by agricultural management practices. Applied Soil Ecology.

[j_jofnem-2022-0046_ref_038] Rosenberg R. (1976). Benthic faunal dynamics during succession following pollution abatement in a Swedish estuary. Oikos.

[j_jofnem-2022-0046_ref_039] Schafer C. T. (1973). Distribution of foraminifera near pollution sources in Chaleur Bay. Water, Air, and Soil Pollution.

[j_jofnem-2022-0046_ref_040] Schumacher L. A., Grabau Z. J., Wright D. L., Small I. M., Liao H. (2020). Nematicide influence on cotton yield and plant-parasitic nematodes in conventional and sod-based crop rotation. Journal of Nematology.

[j_jofnem-2022-0046_ref_041] Sieriebriennikov B., Ferris H., de Goede R. G. M. (2014). NINJA: An automated calculation system for nematode-based biological monitoring. European Journal of Soil Biology.

[j_jofnem-2022-0046_ref_042] Smolik J. D. (1983). Effect of nematicide treatments on nontarget nematode populations associated with corn. Plant Disease.

[j_jofnem-2022-0046_ref_043] Sylvia D., Alagely A., Chellemi D., Demchenko L. (2001). Arbuscular mycorrhizal fungi influence tomato competition with bahiagrass. Biology and Fertility of Soils.

[j_jofnem-2022-0046_ref_044] Trap J., Bonkowski M., Plassard C., Villenave C., Blanchart E. (2016). Ecological importance of soil bacterivores for ecosystem functions. Plant and Soil.

[j_jofnem-2022-0046_ref_045] Van den Hoogen J., Geisen S., Routh D., Ferris H., Traunspurger W., Wardle D., Goede R., Adams B., Ahmad W., Andriuzzi W., Bardgett R., Bonkowski M., Campos-Herrera R., Cares J., Caruso T., Caixeta L., Chen X., Costa S., Creamer R., Crowther T. (2019). Soil nematode abundance and functional group composition at a global scale. Nature.

[j_jofnem-2022-0046_ref_046] Waldo B. D., Grabau Z. J., Mengistu T. M., Crow W. T. (2019). Nematicide effects on non-target nematodes in bermudagrass. Journal of Nematology.

[j_jofnem-2022-0046_ref_047] Wei P., Liu Y., Li W., Qian Y., Nie Y., Kim D., Wang M. (2016). Metabolic and dynamic profiling for risk assessment of fluopyram, a typical phenylamide fungicide widely applied in vegetable ecosystem. Scientific Reports.

[j_jofnem-2022-0046_ref_048] Wilson M. J., Kakouli-Duarte T. (2009). Nematodes as environmental indicators.

[j_jofnem-2022-0046_ref_049] Yeates G. W. (1999). Effects of plants on nematode community structure. Annual Review of Phytopathology.

[j_jofnem-2022-0046_ref_050] Zhang K., Schumacher L., Maltais-Landry G., Grabau Z. J., George S., Wright D. L., Small I. M., Liao H. (2022). Integrating perennial bahiagrass into the conventional rotation of cotton and peanut enhances interactions between microbial and nematode communities. Applied Soil Ecology.

[j_jofnem-2022-0046_ref_051] Zhao D., Wright D. L., Marois J. J., Mackowiak C. L., Brennan M. (2010). Improved growth and nutrient status of an oat cover crop in sod-based versus conventional peanut-cotton rotations. Agronomy and Sustainable Development.

